# Scapular Morphometry Informs Suprascapular Nerve Injury Risk During Reverse Shoulder Arthroplasty: A Cadaveric Study

**DOI:** 10.3390/jcm15051927

**Published:** 2026-03-03

**Authors:** Dave Osinachukwu Duru, Salma Chaudhury, Niel Kang, Cecilia Brassett

**Affiliations:** 1 Human Anatomy Centre, Department of Physiology, Development and Neuroscience, University of Cambridge, Cambridge CB2 1TN, UK; 2 School of Clinical Medicine, University of Cambridge, Cambridge CB2 1TN, UK; 3 Department of Trauma and Orthopaedics, Cambridge University Hospitals NHS Foundation Trust, Cambridge CB2 0QQ, UK

**Keywords:** reverse shoulder arthroplasty, suprascapular nerve, scapular dimensions, safe zones, glenoid baseplate, peripheral screw, cadaveric, patient-specific

## Abstract

**Background**: Reverse shoulder arthroplasty (RSA) relies on secure baseplate fixation to the glenoid. This carries a risk of suprascapular nerve (SSN) injury during peripheral screw insertion. Although fixed safe zones have been described, it remains unclear whether these scale with scapular morphometry or whether common screw positions confer differential SSN risk. **Methods:** Twenty cadaveric shoulders (ten pairs) were dissected. The superior safe zone (distance from the supraglenoid tubercle to SSN at the suprascapular notch) and posterior safe zone (distance from the glenoid rim to SSN at the spinoglenoid notch) were measured. Scapular dimensions (height, spine length, width) were measured. In ten shoulders, simulated RSA baseplate fixation was performed with superior screws placed at 11, 12, or 1 o’clock and posterior screws at 8, 9, or 10 o’clock. Screw lengths were based on glenoid depth. Cortical breach and SSN proximity were recorded. Linear regression assessed relationships between scapular dimensions and safe zones. **Results:** The superior safe zone (mean 2.9 ± 0.5 cm) significantly correlated with scapular dimensions (r = 0.78–0.86; *p* < 0.0001). All superior screws remained intraosseous across configurations. The posterior safe zone (1.9 ± 0.6 cm) showed no correlation. Posterior cortical breach occurred in 50% of specimens across all tested positions and was associated with smaller scapular spine length (*p* = 0.027). No significant difference in SSN proximity was observed between posterior screw positions. **Conclusions:** Scapular dimensions predict the superior, but not posterior, safe zone. Scapulae with shorter spine lengths demonstrated increased risk of posterior cortical breach, independent of screw position. These findings establish anatomical scalability of the superior safe zone and suggest that scapular morphometry may inform preoperative RSA planning; however, prospective validation is needed before routine clinical implementation.

## 1. Introduction

Reverse shoulder arthroplasty (RSA) is an effective treatment for rotator cuff tear arthropathy and comminuted proximal humeral fractures [1,2,3]. Despite often improving pain and function, RSA is associated with recognized complications; these include glenoid loosening, postoperative fracture, periprosthetic infection, and neurovascular injury [4,5,6]. Among these, suprascapular nerve (SSN) injury is particularly serious, most commonly occurring during glenoid baseplate drilling and peripheral screw insertion. Superior and posterior screws that penetrate beyond the glenoid pose the greatest risk, given their close anatomical relationship to the SSN [7,8,9,10]. Clinically, SSN injury may manifest as postoperative pain and weakness, notably reduced external rotation [9,11].

To mitigate this risk, prior studies have proposed fixed anatomical “safe zones” to guide drilling and screw placement during RSA [12,13]. These values are typically derived from minimum distances measured within study cohorts and are intended to provide population-based thresholds for surgical safety. However, these safe zones do not account for inter-individual variation in scapular morphometry, limiting their ability to scale with patient-specific anatomy.

One dry bone study demonstrated that the superior, but not posterior, safe zone in RSA correlates with scapular height and width [14]. Whether these relationships persist in intact specimens with preserved neurovascular anatomy remains unknown. Furthermore, no prior study has evaluated the relationship between these safe zones and scapular spine length, a scapular dimension that is readily measurable and may offer a practical means of personalizing surgical planning [15,16].

Peripheral screw positioning during RSA is guided largely by biomechanical considerations, with comparatively limited evaluation of neural risk. Current convention favors placement of the superior screw at the 1 o’clock position of the glenoid, targeting the base of the coracoid process, to optimize fixation, with the posterior screw typically placed at 10 o’clock [8,10,17]. However, it remains unclear whether alternative superior screw positions at 11 or 12 o’clock, or posterior positions at 8 or 9 o’clock, confer differential risk to the SSN.

Accordingly, the aims of this cadaveric study were to (1) quantify the superior and posterior safe zones and assess their relationships with scapular dimensions in intact specimens, and (2) evaluate cortical breach risk and SSN proximity with commonly used superior and posterior baseplate screw positions, with particular attention to the influence of scapular size.

## 2. Materials and Methods

### 2.1. Specimen Preparation

Ten pairs of cadaveric shoulder specimens (*n* = 20) were provided by the Human Anatomy Centre within the Department of Physiology, Development and Neuroscience, University of Cambridge, UK. Prior to their deaths, the donors (5 males and 5 females) had provided consent to be involved in anatomical research, in compliance with the Human Tissue Act (2004). The donors were of White British descent, with a mean age at time of death of 80.8 ± 8.9 years. The donors were preserved by cannulation of the femoral or common carotid artery, and pressurized injection of a solution containing 38% ethanol, 4.2% formaldehyde, 1.5% methanol, and 56.3% distilled water. None of the donors had undergone previous shoulder surgery.

### 2.2. Measurements

Dissection was performed via an open approach. Skin and subcutaneous tissue over the superior and posterior shoulder were removed. Trapezius was incised along its insertions (on the clavicle, scapular spine, and acromion) and reflected laterally to expose the underlying supraspinatus. Deltoid was incised along its insertions (on the scapular spine and acromion) and reflected anterolaterally to expose infraspinatus. Supraspinatus was incised medially and reflected posterolaterally from the supraspinous fossa. Further dissection exposed the supraglenoid tubercle and SSN at the suprascapular notch. Infraspinatus was incised medially and reflected laterally from the infraspinous fossa. Further dissection exposed the glenoid rim and SSN at the spinoglenoid notch.

**Figure 1:**
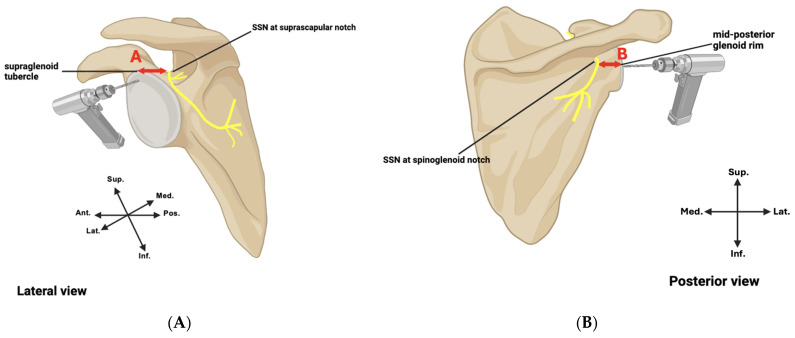
Safe zones. Schematic figures of the (**A**) lateral view of the left scapula and (**B**) posterior view of the right scapula, with the suprascapular nerve (SSN) indicated in yellow. (**A**) = superior safe zone. (**B**) = posterior safe zone. Power drills (not drawn to scale) reflect the typical sites of drilling to enable peripheral screw placement.

**Figure 2:**
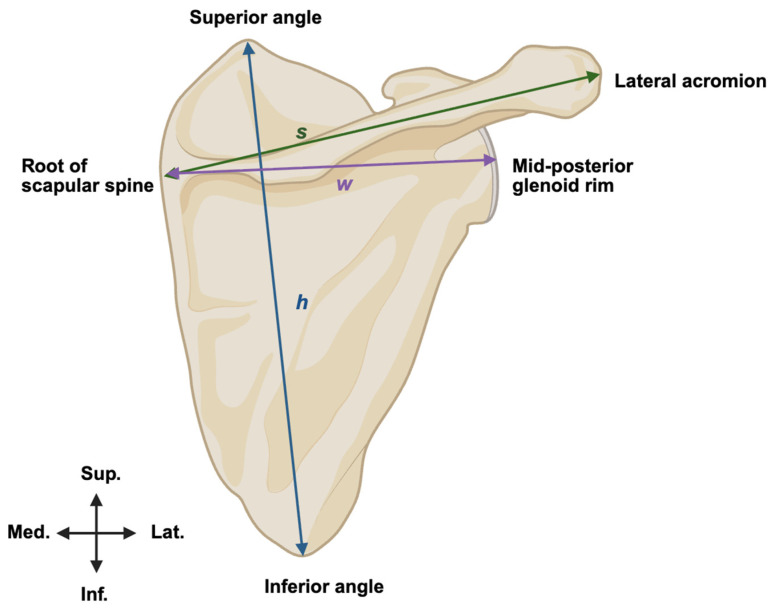
Measurement of scapular dimensions. Posterior view of right scapula. (h) = height, (s) = spine length, (w) = width.

The superior safe zone was measured as the distance from the supraglenoid tubercle to the SSN at the suprascapular notch. The posterior safe zone was measured as the distance from the mid-aspect of the posterior glenoid rim to the SSN at the spinoglenoid notch (Figure 1). Scapular dimensions (height, spine length, and width) were measured (Figure 2). All measurements were initially acquired to 0.1 mm using digital calipers and ImageJ v1.54g software. Scapular dimensions and safe zone measures were converted to centimeters and reported to one decimal place to reflect clinically meaningful resolution. To ensure reliability, measurements were performed in triplicate by a single observer and verified by an independent observer, with inter-observer correlation > 0.95.

### 2.3. Simulated Baseplate Fixation

**Figure 3:**
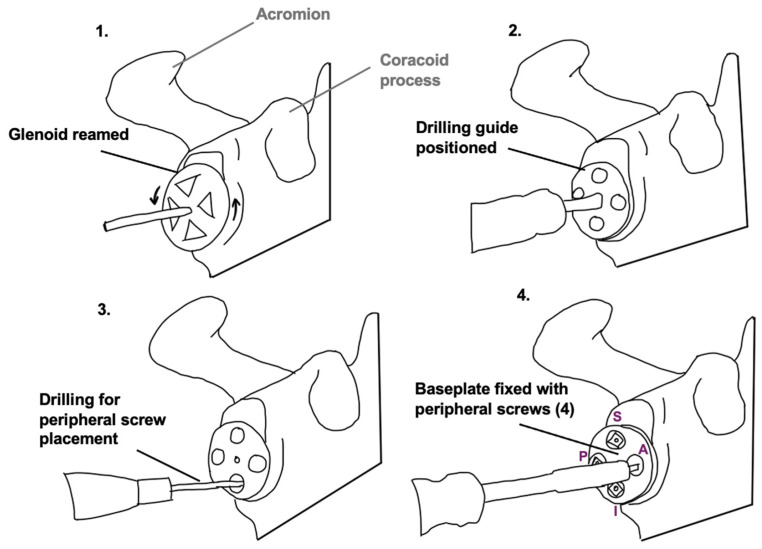
Stages of glenoid preparation for baseplate fixation in RSA (1–4). S = superior screw; P = posterior screw; A = anterior screw; I = inferior screw; Note. Only superior and posterior screws were inserted in this study.

The baseplate fixation aspect of RSA was performed on ten shoulder specimens, using Stryker Tornier Perform^®^ Reversed Glenoid surgical equipment (Portage, MI, USA). The procedures were performed by consultant orthopedic surgeon senior authors (S.C. and N.K.). Dissection was performed to expose the glenohumeral joint, and the humeral head was disarticulated. The accessed glenoid was reamed and the baseplate was fixed to the glenoid by drilling, followed by superior and posterior screw placement (Figure 3). Screw lengths were determined via depth gauge measurements.

**Figure 4:**
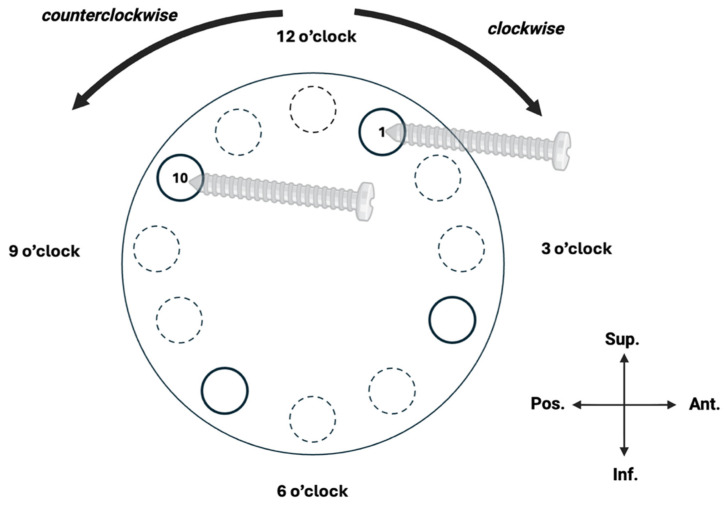
The glenoid as a clock face, used to guide peripheral screw placement in RSA baseplate fixation (exemplified by right glenoid).

Initially, the baseplate was fixed with the superior screw placed at the 1 o’clock position (targeting the base of the coracoid) and the posterior screw at 10 o’clock (Figure 4). Where a screw breached the glenoid cortex, the distance from the screw tip to the SSN was measured. Following these measurements, the baseplate and screws were repositioned within the same specimen by rotating the construct 30° counterclockwise, relocating the superior screw to the 12 o’clock position and the posterior screw to 9 o’clock. The same measurements were repeated. A further 30° counterclockwise rotation was then performed, positioning the superior screw at 11 o’clock and the posterior screw at 8 o’clock, after which the same measurements were again obtained. The order of screw position testing was fixed to allow within-specimen comparison.

### 2.4. Statistical Analysis

Statistical analyses were performed using Microsoft Excel v16.100 and Prism v10.4.2 (GraphPad). Sample size is *n* = 20 for the safe zone analysis, and *n* = 10 for the simulated RSA baseplate fixation. To test for normal distribution, Q-Q plots and the Shapiro–Wilk test were employed [18]. Simple linear regression was used to test for correlation between different variables. The correlation strength was assessed using Pearson’s r and R^2^ values. The F-test was used to investigate for significant, non-zero relationships between variables. The definition of statistical significance was *p* ≤ 0.05.

## 3. Results

### 3.1. Safe Zones

The mean superior safe zone was 2.9 ± 0.5 cm (range: 2.2–3.7 cm). The mean posterior safe zone was 1.9 ± 0.6 cm (1.2–3.0 cm). There was no significant difference between left and right shoulders from the same donors (two-tailed paired *t*-test, *p* > 0.05).

The superior safe zone significantly correlated with measured scapular height, spine length, and width (Table 1). Scapular height was the best predictor of the superior safe zone (r = 0.86; *p* < 0.0001), followed by spine length (r = 0.82; *p* < 0.0001) and width (r = 0.78; *p* < 0.0001) (Table 2). The posterior safe zone did not correlate with scapular dimensions. This supports the use of scapular morphometry to scale the superior safe zone, but not the posterior safe zone.

### 3.2. Screw Analysis

In each specimen, superior and posterior screws were inserted to fix the baseplate to the glenoid. The mean superior screw length was 29.6 ± 4.8 mm (22–37 mm). The mean posterior screw length was 20.6 ± 2.5 mm (18–24 mm). The superior screw remained intraosseous at all three positions within the same specimen (11–1 o’clock) in all ten shoulders. The posterior screw was intraosseous at all positions (8–10 o’clock) in five shoulders but perforated the bone at all three positions in the other five shoulders. In the five shoulders where the posterior screw perforated the glenoid, no significant differences were observed in SSN injury risk (as indexed by the distance of the screw tip to the SSN) across the three positions (Table 3). The five breached scapulae had significantly smaller spine lengths (unpaired *t*-test; *p* = 0.027) but did not differ in height (*p* = 0.299) or width (*p* = 0.075).

## 4. Discussion

This cadaveric study supports patient-specific baseplate screw placement and drilling in RSA, guided by measurable scapular morphometry.

The superior safe zone demonstrated strong correlation with all three scapular dimensions; this suggests the superior safe zone may be reliably predicted by scapular morphometry. This extends prior dry bone observations, where scapular height and width correlated with the superior safe zone [14]. The present study differed in that it found a novel correlation between the superior safe zone and scapular spine length. Additionally, instead of dry bone, the present study utilized intact cadaveric specimens, with SSN present. These relations support the use of bespoke safe operative zones, informed by scapular dimensions, to guide RSA.

In this study, superior screw trajectory did not risk the SSN. Superior screws at 11, 12, or 1 o’clock remained intraosseous, out of the SSN course. This suggests that deviation from the conventional preference for 1 o’clock, targeting the coracoid base, does not alter SSN risk. Surgeons may therefore exercise flexibility in superior screw position, prioritizing available bone stock and construct stability without compromising nerve safety, provided screw length is appropriate.

Conversely, the posterior safe zone showed no relationship with scapular dimensions, similarly observed in the dry bone study [14]. Posterior cortical breach occurred in half of the specimens across tested trajectories (8–10 o’clock). No position conferred a measurable safety advantage. The only parameter associated with increased breach risk was smaller scapular spine length, suggesting a high-risk group. Hence, increased caution should be had when placing posterior screws, particularly in morphometrically smaller scapulae. While no screw contacted the SSN, cortical breach would place the SSN at increased risk of irritation or neuropathy via postoperative traction or scarring [5,19]. However, this increased risk remains speculative, and interpretations are limited by the small sample size, as just five specimens exhibited cortical breach.

Furthermore, the mean and minimum safe zone distances found in this study align with the prior literature [12,13,20,21], reinforcing the validity of our observations. However, while most earlier studies conceptualized safe zones as definite, our data demonstrate scalability, specifically of the superior safe zone.

Furthermore, the findings of no direct SSN impingement with screw placement contrast with prior cadaveric reports, which found high rates of SSN contact during simulated RSA [7,8]. This discrepancy is likely attributable to differences in screw length selection (Table 4).

Earlier investigations employed uniformly longer screws than the present study. As such, SSN contact rates in these studies may overestimate clinical risk when conservative screw lengths are used.

Collectively, these findings support a scalable superior safe zone informed by scapular size. Posterior fixation requires individualized risk assessment irrespective of clock-face position, with careful attention to scapular spine length and available bone stock. Navigation-assisted and CT-based planning techniques have optimized screw placement and may facilitate improved bony fixation through longer screw lengths [22,23,24,25]. However, incorporation of scapular morphometry and the safe zone measures may provide a complementary anatomical framework to support neurovascular safety. Particularly for the posterior screw, which did not demonstrate predictable scaling with scapular morphometry, CT-based preoperative planning, patient-specific instrumentation, or navigation-assisted placement may be important to optimize trajectory and minimize cortical breach risk.

Despite the strengths of the present study, with its rigorous measurements and novel correlations, there are several limitations to address. For instance, the study’s sample size was small, which may raise concerns regarding external validity and robustness. Also, the study sample comprised only elderly White British donors, which may limit generalizability; however, this age demographic closely reflects the typical RSA patient cohort. Embalmed specimens may exhibit mild soft-tissue shrinkage compared with in vivo anatomy, though this is unlikely to meaningfully alter bone-nerve spatial relationships. Also, all procedures were performed by experienced, fellowship trained shoulder surgeons, which may potentially underestimate the risks associated with less experienced operators. Additionally, only a single company implant was evaluated, and screw trajectory may be influenced by baseplate shape, glenoid morphology, and degenerative wear patterns, typical of RSA patients. Given our use of cadaveric specimens without evident shoulder pathology, the clinical translation of the findings is limited, given the challenges posed by deformed glenoids.

Future research should validate the anatomical relationships between scapular dimensions and RSA safe zones via 3-D CT scan-based measures and measurements in live patients. Additionally, we recommend prospective studies integrating scapular dimensions into preoperative RSA planning, with evaluation of the effect on safety, operating time, and patient outcomes. Investigation across diverse ethnic populations and utilizing differing implant constructs may enhance the global applicability of our findings.

## 5. Conclusions

In this cadaveric study, scapular dimensions predicted the superior safe zone for RSA, demonstrating anatomical scalability. In contrast, the posterior safe zone did not scale with scapular morphometry. Posterior cortical breach occurred in 50% of specimens, particularly in shoulders with shorter scapular spine lengths. These findings suggest that superior screw planning may be anatomically informed by scapular dimensions, while reliance on anatomy alone may be insufficient for posterior screw planning; however, the clinical effectiveness of morphometry-guided planning requires prospective validation using CT-based assessment and outcome studies.

## Figures and Tables

**Table 1:** Measurement of scapular dimensions.

	All Specimens	Left Side	Right Side
Height	14.6 ± 1.5 (11.1–16.7)	14.8 ± 1.6	14.4 ± 1.5
Spine length	14.0 ± 1.4 (11.4–16.1)	13.8 ± 1.6	14.3 ± 1.2
Width	10.8 ± 1.2 (8.5–13.0)	10.7 ± 1.2	10.9 ± 1.3 ^1^

^1^ All measurements are in cm (mean ± SD). The range is indicated in brackets.

**Table 2:** Correlations between scapular dimensions and study measures.

Safe Zone vs. Scapular Dimension	Line of Best Fit Equation	R^2^	*p*-Value
Superior vs. height	Y = 0.0271X − 1.00	0.733	<0.0001
Superior vs. spine length	Y = 0.0275X − 0.91	0.668	<0.0001
Superior vs. width	Y = 0.0300X − 0.28	0.601	<0.0001
Posterior vs. height	Y = −0.00736X + 2.96	0.0400	0.398
Posterior vs. spine length	Y = 0.00370X + 1.37	0.00894	0.692
Posterior vs. width	Y = 0.0116X + 0.64	0.0663	0.273 ^1^

^1^ Superior = superior safe zone distance; Posterior = posterior safe zone distance; Y = safe zone distance in centimeters; X = scapular dimension (height/spine length/width) in centimeters.

**Table 3:** Proximity of perforated posterior screw tips to the SSN.

Screw Position	Distance Mean (SD), cm
10 o’clock	1.0 (0.3)
9 o’clock	1.2 (0.3)
8 o’clock	1.3 (0.5)
*p*-value = 0.9167 (n.s.) ^1^	

^1^ n.s. = One-way repeated measures ANOVA; *p* > 0.05.

**Table 4:** Comparison of screw lengths between the present study and the literature.

	Present Study	Molony et al. [7]	Vance et al. [8]
Superior screw	29.6 ± 4.8 (22–37)	36.6 (32–42)	44
Posterior screw	20.6 ± 2.5 (18–24)	26.2 (18–32)	44 ^1^

^1^ All measurements are in mm (mean ± SD). The range is indicated in brackets.

## Data Availability

The raw data supporting the conclusions of this article will be made available by the authors on request.

## Abbreviations

The following abbreviations are used in this manuscript:

RSA	Reverse shoulder arthroplasty
SSN	Suprascapular nerve
CT	Computed tomography
